# ASO Author Reflections: Centralizing Esophagogastric Cancer Surgery: Beyond Volume Thresholds

**DOI:** 10.1245/s10434-026-19714-x

**Published:** 2026-04-30

**Authors:** Cezanne D. Kooij, Irene S. Zuin, Lucas Goense

**Affiliations:** University Medical Center Utrecht, Utrecht, the Netherlands

Surgery remains the cornerstone in the management of esophagogastric cancer, yet these complex procedures continue to be associated with considerable morbidity and mortality.^1,2^ For decades, higher hospital volume has been associated with improved outcomes after complex surgical procedures. Nevertheless, the evidence base remains fragmented due to heterogeneity in study designs, variation in outcome measures, and inconsistent definitions of what constitutes a “high-volume” center. As a result, centralization policies vary widely and no universally accepted volume thresholds exist.^3^

In this comprehensive meta-analysis, we evaluated recent global evidence on the association between hospital volume and outcomes after esophagectomy and gastrectomy.^4^ Our findings demonstrate that higher hospital volume is consistently associated with improved outcomes, including lower postoperative mortality, reduced complication rates, shorter length of hospital stay, and improved long-term survival. Importantly, we identified indicative procedural volume thresholds (43 cases for esophagectomy and 15 cases for gastrectomy annually), beyond which the association between procedural volume and 30-day mortality plateaus. These values exceed many currently used policy benchmarks and may help inform further centralization efforts. At the same time, they should not be interpreted as rigid cutoffs. The included studies were heterogeneous, publication bias was present, and adjustment for case complexity was often limited.

Looking ahead, future research should move beyond volume alone. Hospital volume is likely a surrogate for broader institutional strengths, including experienced multidisciplinary teams, specialized perioperative care pathways and nursing, and the ability to rapidly detect and treat complications. A more detailed understanding and quantification of these factors will be essential to refine centralization strategies and ensure that improvements in patient outcomes are driven not only by higher case volumes, but also by the quality and organization of care.
